# 4th International Symposium of Advanced Topics in Exercise Physiology: Non-pharmacological treatment for the improvement of the quality of life in the Elderly: Virtual. 3-5 November 2020

**DOI:** 10.1186/s12919-020-00205-0

**Published:** 2020-12-23

**Location and date:** Ensenada Baja California México 3rd -5th November 2020 (virtual symposium).

**Sponsorship:** Publication costs for this supplement were funded by the Consejo Nacional de Ciencia y Tecnología (CONACYT) México.

**Organizer:** Facultad de Deportes Campus Ensenada, Universidad Autónoma de Baja California

**Conference website:** https://sitafe2020.webnode.es/

**List of editors**

**Table:** 

Full name of editor	Institution
Patricia C. García Suárez	Facultad de Deportes Campus Ensenada, Universidad Autónoma de Baja California. México
Iván Rentería	Facultad de Deportes Campus Ensenada, Universidad Autónoma de Baja California. México
Ermilo Canton Martínez	Facultad de Deportes Campus Ensenada, Universidad Autónoma de Baja California. México
José Moncada-Jiménez	Human Movement Sciences Research Center (CIMOHU), University of Costa Rica. Costa Rica
Luis M. Gómez-Miranda	Facultad de Deportes Tijuana, Universidad Autónoma de Baja California. México
Melinna Ortiz-Ortiz	Facultad de Deportes Tijuana, Universidad Autónoma de Baja California. México
Jorge A. Aburto-Corona	Facultad de Deportes Tijuana, Universidad Autónoma de Baja California. México
Alberto Jiménez Maldonado	Facultad de Deportes Campus Ensenada, Universidad Autónoma de Baja California. México

## S1 Correlation of body weight with bone density of lower limbs in older adult women

### Rosa María Cruz Castruita, Ernani Francesco Cátalan Bibene, Ricardo López García

#### Facultad de Organización Deportiva, Universidad Autónoma de Nuevo León, Nuevo León, México

##### **Correspondence:** Ricardo López García (ricardo78-82@hotmail.com)

**Background**

Being overweight can be a factor to benefit bone density, although in the postmenopause stage, women with high body weight tend to suffer from weakness in the bones of the lower limbs region due to low levels of estrogens, hormones that help mineralize bone. The objective of this study was to correlate body weight with lower limb bone mineral density in older adult women.

**Materials and methods**

110 women (66.08 ± 4.36 years of age), retired from an educational institution participated. In which weight (kg) and height (cm) were evaluated with the ISAK protocol, bone mineral density (g / cm2) was also assessed with the dual x-ray absorptiometry (DEXA) equipment, measuring the right and left side of the trochanter, femoral neck and leg regions, to later obtain the mean value of both sides. The SPSS program with Pearson's test was used to correlate body weight with bone mineral density in the regions evaluated. The level of significance used was *p* ≤.05 and *p* ≤.01.

**Results**

Low associations were found, although with low but significant associations were found of the femoral neck (*r* = 0.244; *p* <.010), trochanter (*r* = 0.274; *p* <.004) and legs (*r* = 0.240; *p* < .012).

**Conclusions**

Although it to preferred that excess weight corresponds to a high amount of lean mass than fat mass, having a high weight can strengthen the density of the bone in the lower regions, this by increasing the mechanical load on the bone, as long as when other factors that can damage the mineral in the bone are considered, such as diet, sedentary lifestyle, high consumption of alcohol and tobacco, and hormonal control.

## S2 Elaboration of food supplements for elderly people with nutritional deficiencies

### Rosalía Reynoso Camacho, Eduardo Castaño Tostado, Minerva Ramos Gómez, Silvia Lorena Amaya Llano, Iza Fernanda Pérez Ramírez, Gloria Andrea Pérez Álvarez

#### Department of Research and Postgraduate Studies in Food, Faculty of Chemistry, Autonomous University of Queretaro, Queretaro, Mexico

##### **Correspondence:** Gloria Andrea Pérez Álvarez (gperez15@uabc.edu.mx)

**Background**

Elderly people have deficiencies of protein, dietary fiber and micronutrients. There is also a high prevalence of diabetes, overweight and obesity, which increase these deficiencies. Food supplements are an alternative to reduce the effects of these alterations. In the national market, food supplements do not satisfy a nutritional and sensory quality to be accepted by the consumer. That is why the objective of this work was to develop food supplements with high nutritional quality for the elderly with different conditions that complement their daily needs for protein, fiber and micronutrients.

**Materials and methods**

The supplements were elaborated with whey protein concentrate, polydextrose and a mix of micronutrients. A design of mixtures with extreme vertices was used, varying the content of protein, fiber and micronutrients in each formulation. It was evaluated for its solubility index and viscosity, those were factors to select 4 formulations with better palatability (one for each health status), and then these were characterized with proximal, sensory and stability tests.

**Results**

The solubility index was higher by 461% and the viscosity was similar to commercial brands; in the proximal test, it obtained high values in proteins (27-60.9% vs 15% of the commercial brand) and fiber (10-23.3% vs. 6%). The sensory test suggests that new formulations were superior to the commercial sample in texture and flavor. Finally, a stability study was carried out at 25, 35 and 45 ºC. At 45ºC and 90 days of storage, a slight yellow color (b *) was observed in all food supplements, no protein oxidation was observed, and only a decrease in protein digestibility was observed at 45ºC in high protein supplements.

**Conclusions**

The use of alternative ingredients and flavor enhancers allowed the elaboration of new nutritional food supplements for elderly people with nutritional deficiencies with good functional parameters and sensory acceptability.

## S3 Neck circumference as an indicator of malnutrition in the elderly

### Alondra García-Ibáñez, Sugey Chávez-Herrera, Fátima López-Alcaraz, Jaime Alberto Bricio-Barrios

#### Faculty of Medicine, Universidad de Colima, Colima, Mexico

##### **Correspondence:** Jaime Alberto Bricio-Barrios (jbricio@ucol.mx)

**Background**

It is essential to detect nutritional deficiencies in older adults using simple tools such as neck circumference. This study aimed to compare neck circumference in older adults with and without malnutrition in Colima, Mexico.

**Materials and methods**

In this cross sectional study, 293 older adults were included, which were divided into three study groups: (1) with undernutrition: (evaluated with body mass index (BMI) <23 kg/m^2^, tricipital skinfold <P10, arm circumference <24 cm and calf <31 cm); (2) the overweight and obesity group (evaluated with BMI >28 kg/m^2^, body fat and high waist circumference) and (3) the control group (with 23 to 28 kg/m^2^ of BMI and adequate fat mass); In all groups, the neck circumference was compared, which was taken with the person sitting, with the head in a neutral position and looking straight ahead, using a metal tape that passed through the neck over the thyroid cartilage. we analyzed the results as mean and standard deviation, and one-way ANOVA and Tukey test (*post* hoc) as an inferential analysis.

**Results**

71% (n= 208) were women and 76.8% (n= 225) belong to a recreational group; 53.9% (n= 158) lived with a family member, 13.7% (n= 40) lived alone and 32.4% (n= 95) live with a caregiver. Neck circumference showed significantly lower values ​​in the group with malnutrition (n= 43 [33.68 ± 2.56 cm]) compared to the control group (n= 134, [35.66 ± 3.14 cm]) and the overweight and obesity group (n= 116 [38.04 ± 3.59 cm]) (p <0.0001).

**Conclusion**

Older adults with undernutrition present statistically lower neck circumference compared to those who are overweight and obese, which sets a guideline in future studies on the importance of measuring neck circumference as a complement for the detection of malnutrition.

## S4 Predictive equation that determines health costs in institutionalized older adults through selective screening: a pilot study

### Karen Jovana García-Nevarez, Jaime Alberto Bricio-Barrios

#### Faculty of Medicine, Universidad de Colima, Colima, Mexico

##### **Correspondence:** Jaime Alberto Bricio-Barrios (jbricio@ucol.mx)

**Background**

Older adults have become a priority in public health, constituting an age group that currently receive costly curative care and generally do not receive prevention services. We can promote an early detection of a certain disease in order to improve its prognosis and avoid premature morbidity and mortality in this age group. The aim of this study was to establish a predictive equation through the multivariate linear model that determines health costs from selective screening and anthropometric parameters.

**Material and methods**

a cross-sectional study in adults older than 60 years and older, institutionalized in Colima, Mexico (n= 26). The multivariate linear model was used to develop the equation, Barthel index, MNA, Share-Fi scale and anthropometric measurements were made in each participant.

**Results**

The costs in the health of the elderly can be predicted by 31.8% through the following equation (r = 0.583; p <0.001): *Health costs (in Mexican pesos)= 16207.1 + (89.8 x arm circumference [cm]) - (461.2 x calf circumference [cm]) - (123.8 x waist circumference [cm]) + (555.9 x body mass index [kg/m*^*2*^*]) - (35.3 x right hand strength [kg]) + (1079.8 x sex [1=female, 2=male]) - (1.7 x Barthel index) - (114.8 x MNA score) - (230.4 x share-fi score)*. The study showed that the variable that most influences health costs, which is the Barthel index (r = -0.338, p = 0.025).

**Conclusions**

The application of the proposed predictive equation could become a quick and easy way to determine disease early to improve the prognosis of the patient and therefore the institution would provide immediate care.

## S5 Food consumption in Cuban older adults according to cognitive disorders

### Yeneisy Lanyau Domínguez^1^, Armando Rodríguez Suárez^1^, Ramón Suárez Medina^1^, Consuelo Macías Matos^1^, Juan de Jesús Llibre Rodríguez^2^, María Elena Díaz Sánchez^1^, María Eugenia Quintero Alejo^1^

#### ^1^Center of Nutrition and Food Hygiene, National Institute of Hygiene, Epidemiology and Microbiology, Havana, Cuba; ^2^Alzheimer Studies Center, Havana University of Medical Sciences, Havana, Cuba

##### **Correspondence:** Yeneisy Lanyau Domínguez (ylanyau@infomed.sld.cu)

**Background**

The dietary pattern has been studied in individuals with dementia in different epidemiological studies, but results were not been consistent. The objective is to determine differences in food consumption in individuals according to cognitive impairment and to identify the association between the inadequate intake of different food groups with cognitive disorders in Cuban older adults.

**Materials and Methods**

A cross-sectional analytical study was carried out on 424 adults over 65 years of age; 43 with Alzheimer's disease (AD), 131 with Mild Cognitive Impairment (MCI), and 250 individuals without cognitive impairment, all of them from Havana City. Dementia was diagnosed using criterion 10/66 and DSM-IV and for the DCL Petersen criterion. Diet was evaluated through a weekly frequency of consumption survey of the main food groups. Kruskal Wallis, chi-square test of homogeneity, and the prevalence ratio were used for statistical analysis.

**Results**

The pattern of food consumption was similar between groups of individuals according to cognitive decline. Dairy (4.19; 4.04-4.34) and cereals (4.17; 4.07-4.28) appear as the food groups with the highest weekly consumption frequency, equivalent to 6 days a week. Followed by vegetables (3.39; 3.22- 3.56), sugar (3.38; 3.19-3.57), and meats (3.23; 3.09-3.38) that were consumed between 4-5 days a week. Grains (2.90; 2.74-3.05), fruits (2.77; 2.60-2.93), and fats (2.73; 2.54-2.92) were consumed 2-3 days a week. Fish was about 1 time per week (0.82; 0.73-0.91). Sufficient consumption of cereals and viands was significantly lower in the EA group (p<0.05), while for meat it was significantly higher in this group (p<0.05).

**Conclusions**

The found pattern of food consumption was independent of the cognitive status of the subject. The insufficient intake of cereals and viands was inversely associated with AD, while the consumption of meats was positively associated with AD.

## S6 Time course of concurrent training performed at moderate intensity on body composition and physical fitness in overweight Mexican older adults

### Ermilo Canton Martínez^1^, Edna Nallely Méndez Hernández^2^, Gabriela Valles Verdugo, Patricia C. García Suárez^1^, Iván Rentería^1^, Alberto Jiménez-Maldonado^1^

#### ^1^Facultad de Deportes Campus Ensenada. Universidad Autónoma de Baja California, Baja California, México; ^2^Departamento de Atención Médica. Instituto de Seguridad y Servicios Sociales de los Trabajadores del Estado. Delegación Baja California, México

##### **Correspondence:** Alberto Jiménez-Maldonado (jimenez.alberto86@uabc.edu.mx)

**Background**

The concurrent training (CT) is an exercise modality that include aerobic and strength exercises. In regarding with the “incompatibility” among endurance and strength training, the CT is perceived as a controversial training. We assess the impact of CT in overweight elderly population

**Materials and methods**

9 elderly-healthy participants (7: females; 2 males) (66.2 ± 4.8 yrs.; BMI: 27.5 ±3.4 kg/m²) were recruited in this study. One week prior to the CT, the senior fitness test (SFT) was applied and body composition (BC) was analyzed (bio-impedance). The CT consisted in sessions that included 10 min of warm-up, 20 min of aerobic exercise (walk, dancing and step up aerobics), 15 min of strength workouts (elastic bands), and 10 min of recovery (flexibility). The length of CT was 9 weeks (3 times/week). The Borg 20 scale was used to regulate the exercise intensity in each session. 72 hrs. after the last CT session, the SFT was applied, moreover the BC determined. Follow up two months of completed the CT, the SFT and BC was measured again.

**Results**

The time up and go was not modified throughout the study (F (3,32);1.503, P=0.2). Likewise, the CT did not modify significantly the aerobic capacity (F (3,32);1.27P=0.3). Opposite, the leg strength was improved by the CT F= 3.35 (3,32), P=0.03). Particularly, the upper body strength was improved by CT, and these effects disappear with detraining (F= 5.54 (3,32), P=0.0035). The BC did not change throughout the study.

**Conclusions**

The CT applied in the present study improved the strength performance in the participants. However, the same program did not impact significantly in the muscle mass. These data suggest the presence of neuromuscular adaptations. Contrary, the CT did not modify the aerobic capacity that suggest a possible “interference effect” among strength and endurance adaptations.

## S7 Body composition and physical performance in Cuban older adults, institutionalized, and tai chi chuan practitioners

### María Elena Díaz-Sánchez, Yeneisy Lanyau-Domínguez, Elisa Llera-Abreu

#### Nutrition Center. National Institute of Hygiene, Epidemiology and Microbiology. Cuba

##### **Correspondence:** María Elena Díaz-Sánchez (maryelends@gmail.com)

**Background**

Aging is associated with profound changes in body composition in late life: adiposity increases and skeletal muscle mass is reduced. This loss with age leads to sarcopenia, which is a multifactorial process including physical inactivity in its development. Sarcopenia establishes its symptoms in inactive individuals but is also observed in some who remain active during their lives. Physical exercise Training is favorable to delay sarcopenia, among other factors that improve the quality of life in old age. Objectives: To analyze body composition and physical performance in elderly, institutionalized, and active Tai Chi Chuan practitioners.

**Materials and methods**

A body composition study was performed on 122 individuals, between 60 and 99 years, attending the daycare for the elderly institutions and Tai Chi Chuan practitioners of the National School of Wushu. Weight and height were measured. Fat mass (FM), fat free mass (FFM), total body water (TBW), and muscle mass (SMM) were obtained by bioimpedance. Maximum grip strength (MGS), Gait speed at 6 meters (V6M), the TUG test, and daily living activities, were realized by standardized protocols. Statistical analysis used: the general linear model, principal component analysis, and polynomial logistic regression.

**Results**

The women had higher FM, lower FFM, TBW, SMM, and MGS than men. There is a significant effect of age on weight increase, decrease in height, SMM, and MGS. The FFM and subcomponents TWB and SMM corresponded to performance variables and were more diminished in the institutionalized elderly. The MGS reduced was more representative in institutionalized elderly. Tai Chi Chuan practitioners have significantly more FFM, physical performance, and activities of daily living than older adults who stay in daycare institutions, which have more sedentary behavior, corresponding to sarcopenia.

**Conclusions**

The physical activity of Tai Chi Chuan offers better predictions for body composition to reduce sarcopenia in the elderly.

## S8 Type 2 diabetes did not affect perceived effort during the 6-minute walk test in older adults. A preliminary study

### Víctor R. Herrera Reyes, Giovana Arambula Barba, Concepción D. Juárez Hernandez, Febe Y. Abarca Rivera, Ermilo Canton Martínez, Patricia C. García Suárez, Iván Rentería, Alberto Jiménez-Maldonado

#### Facultad de Deportes Campus Ensenada. Universidad Autónoma de Baja California, Baja California, México

##### **Correspondence:** Alberto Jiménez-Maldonado (jimenez.alberto86@uabc.edu.mx)

**Background**

The type 2 diabetes (T2D) is a metabolic disease that impair the brain function. The last condition could impact on the perception of effort in T2D patients with respect to non-T2D people.

**Materials and methods**

Eleven elder and healthy participants (66.5 ± 5.7 yrs.) and six T2D patients (65.5 ± 3.2 yrs.) performed the 6-minute walk test (6MWT). Before the 6MWT, in both groups, the stride length was measured. After this, bringing a pedometer (Tanita) the participants performed the 6MWT. At the final of field test, the total distance was estimated (stride length x number of steps). Moreover, during the 6MWT, the researchers walked at side of the participants encouraging them walk as fast as they can. During the exercise test, a heart rate monitor (Polar F10), was used to register the heart rate (HR). A hand-held lactometer was used to register the blood lactate at rest and immediately finished the 6MWT. Finally, to assess the perception of effort, the Borg-20 scale was employed

**Results**

Both groups covered similar distance during the 6MWT (healthy: 552.6 ± 92.88 m) vs (T2D: 574.2± 90.43 m) (*p* =0.6). Likewise, there was not statistical differences among the maximal HR achieved at the end of 6MWT (healthy: 135 ± 20 ppm) vs (T2D: 136 ± 14 ppm) (*p* =0.9). In agreement with this, the delta of blood lactate was not different between groups (healthy: 313.3 ± 300 % vs (T2D: 156 ± 106%) (*p* =0.8). Finally, the participants did not show statistical differences in the Borg scale values (healthy: 11.8 ± 2) vs (T2D: 11.1± 3) (*p* =0.5).

**Conclusions**

The data suggest that the T2D did not affect the perception of effort during the 6MWT in elderly people. However, we did not discard the T2D influence to modify the perception of effort during harder and exhaustive exercise evaluations.

## S9 Effect of COVID-19 home confinement on functional capacity in persons with multiple sclerosis

### Luis Andreu-Caravaca^1,2^, Domingo J. Ramos-Campo^2^, Linda H. Chung^3^, Oriol Abellán-Aynés^1,2^, Pedro Manonelles^1^, Jacobo Á. Rubio-Arias^4^

#### ^1^International Chair of Sports Medicine, Faculty of Medicine, Catholic University of Murcia, Murcia, Spain; ^2^Faculty of Sport, Catholic University of Murcia, Murcia, Spain; ^3^UCAM Research Center for High Performance Sport, Catholic University of Murcia, Murcia, Spain; ^4^LFE Research Group, Department of Health and Human Performance, Universidad Politécnica de Madrid, Madrid, Spain

##### **Correspondence:** Luis Andreu-Caravaca (landreu@ucam.edu)

**Background**

Public health recommendations and government actions during the COVID-19 pandemic, including home-confinement, have changed the lifestyle of people. In Spain, the confinement lasted more than two months, which caused a drastic decrease in the levels of physical activity in the population. In persons with multiple sclerosis (pwMS), who benefit from physical exercise to attenuate the progressive loss of different variables such as strength or functional capacity, confinement may have led to a more pronounced worsening of performance in these variables than population without pathologies. Thus, the aim of this study was to analyze the consequences of confinement caused by the COVID-19 pandemic on the neuromuscular performance and functional capacity in Spanish pwMS.

**Materials and methods**

17 pwMS (age: 43.4 ± 10.9 years; weight: 70.1 ± 12.2 kg; height: 167.5 ± 7.1 cm; body mass index: 24.9 ± 3.3 kg/m^2^; Expanded Disability Status Scale: 2.9 ± 1.3 a.u.) participated in the study. The peak rate of force development (RFD_peak_) during isometric knee extension and the Timed-Up and Go Test (TUG) were analyzed pre and post 2-month confinement.

**Results**

RFD_peak_ decreased significantly in the right leg (mean±SD: 2863.5±540.5 vs 2677.8±546.1; p<0.05; Effect Size (ES):0.342) and experienced a trend to decrease in the left leg (2935.8±433.0 vs 2495.1±873.7; p=0.09; ES: 0.364). TUG significantly decreased in performance (11.9±9.8 vs 12.2±9.9; p<0.05; ES=-0.673) after confinement.

**Conclusions**

Based on our results, home confinement resulted in a worsening of neuromuscular performance and functional capacity in pwMS. These decreases may be due primarily to low levels of physical exercise during confinement. In this context, our results suggest that COVID-19 confinement impairs strength and functionality in persons with MS. Therefore, in situations like confinement, performing home-based exercise it should be recommendable, specifically in population with high risk of functional impairment.

## S10 Physical activity in the senior population in Monterrey

### Luis Tomás Ródenas Cuenca^1^, Samantha Medina-Villaneuva^1^, Luis Bojorquez^2^, Minerva Vanegas-Farfano^1^, Erick Cortinas^1^, Roque Pérez^1^, Raul Martinez Reyes^1^

#### ^1^Facultad de Organización Deportiva, Universidad Autónoma de Nuevo León, Nuevo León, México; ^2^Universidad Autónoma de Occidente, Guasave, México

##### **Correspondence:** Luis Tomás Ródenas Cuenca (luis.rodenascn@uanl.edu.mx)

**Background**

In recent decades the senior population has shown significant growth. In 1999, it was estimated that in Mexico citizens over 60 years of age would increase from 5.7 million in 1995 to 6.8 million in 2000. Currently in 2020, there are 407,278 senior adults over 60 in Nuevo León (INEGI, 2010). Elderly individuals experience a significant decrease in their physical abilities and the incidence of chronic health problems such as cardiovascular disease and osteoporosis increases. We aim to describe the variables of physical activity in the senior population in the Monterrey metropolitan area before the COVID-19 pandemic.

**Materials and methods**

The design was descriptive. The sampling was non-probabilistic for convenience, the sample size consisted of 152 participants older than 60 years (M = 79.70; SD = 10.20). The sample consisted of 79 women (52%) and 73 men (48%). The level of physical activity was measured and evaluated with the International Physical Activity Questionnaire, in its abbreviated version.

**Results**

According to the level of dependency, 96% (n: 146) are in the independent categories and 4% (n: 6), in the mild dependency categories, which shows that active older adults mostly present independent classifications for their age. Regarding the level of physical activity, 54% (n: 82) was located within the moderate level; 42% (n: 64) were low or inactive and 4% (n: 6) had a high level of physical activity.

**Conclusions**

This study is part of an investigation where physical activity is also being evaluated during the pandemic, predicting that physical activity will decrease, therefore, it would be very important to generate appropriate interventions of physical activity for this age group and that are guided by qualified professionals and not by “sports” influencers. This study was realized before the he COVID-19 pandemic.

## S11 Anthropometric profile and physical condition of older adults from Armenia, Quindío, Colombia. Preliminary study

### Diana María García-Cardona^1^, Mónica A Ramírez E^1^, Marisel Toro^1^, Oscar Eduardo Sánchez-Muñoz^1^, Miguel Castillo T^1^, Andrés Cadena-Montoya^1^, Valentina Calderón B^1^, Martha N Cardona^1^, Julián Ramírez-Gutiérrez^2^, Pedro Campos R^1^, Cristian R Porras^1^, Hernán Restrepo Ospina^1^

#### ^1^Grupo de Investigación en Fisiología de la Actividad Física y la Salud, Universidad del Quindío, Armenia, Colombia; ^2^Grupo de Investigación en Fisiología de la Actividad Física y la Salud, Universidad Surcolombiana, Neiva, Huila, Colombia

##### **Correspondence:** Diana María García-Cardona (dmgarcia@uniquindio.edu.co)

**Background**

According to the National Department of Statistics, 18% of Colombians are over 60 years old, it is estimated that by 2050 it will increase to 23%. Quindío is the region with the highest rate of aging, and in its capital (Armenia) there are 50,840 older adults. The purpose of the study was to determine the anthropometric profile and physical condition of the older adults in the city of Armenia.

**Materials and Methods**

Cross-sectional, descriptive study in 181 older adults from the city of Armenia (162 women, 19 men). The subjects filled out the informed consent.

The anthropometric variables were age, mass, height, body mass index, waist-hip ratio (WHR), and the physical condition variables were muscular strength, flexibility, aerobic endurance, balance and agility. To determine them, it was used the Senior Fitness Test (SFT).

**Results**

On average, the age was 69.69±7.2 years, height 1.56±7.0 m, body mass 67.2±11.5 kg, BMI 26.72±4.35 kg/m^2^ in women and in men, age 70.78±7.9, height 1.57± 8.5 m, body mass 64.4±9.7kg and BMI 26.93±4.8. On average, the cardiovascular risk, estimated with the WHR, was high in women and low in men.

The physical condition results showed that, on average, men were found within the reference intervals, in flexibility variables, lower body strength (75–79 and 85–89 years), and agility and dynamic balance (65–69 years).

In women, it was found that all the variables were within the SFT reference values at the age of 90–94 years, contrary to what was observed in the age of 60-64 years.

**Conclusions.**

The BMI for both sexes was within the normal range.

Women aged 90-94, and men 75-79 years old presented a better anthropometric profile.

Physical condition was within the reference values in women aged 90-94, and men aged 75-79, as opposed to their counterparts aged 60-64.

## S12 Effect of a multicomponent training on the parasympathetic nervous system in older adults

### Silvia Carolina Medrano Mena, Rosa María Cruz-Castruita, José Raúl Hoyos-Flores

#### Facultad de Organización Deportiva (Universidad Autónoma de Nuevo León, Nuevo León, México)

##### **Correspondence:** Silvia Carolina Medrano Mena (silvia.medranomn@uanl.edu.mx)

**Background**

Heart rate variability (HRV) has been very useful to study the autonomic activity of the heart. However, no evidence has currently been found to demonstrate the effects that a multicomponent modality training has on this variable. Objective: to evaluate the effect of a multicomponent training program on parasympathetic activity in older adults.

**Materials and methods**

Quasi-experiemtal study in a sample of 45 older adults of both genders, with random assignment to the experimental group (EG; *n* = 20 [68 ± 11.97 years]) and control group (CG; *n* = 25 [73 ± 6.85 years]). A baseline measurement was made at three months and six months. A multicomponent training program (strength, endurance, flexibility, coordination and balance) was applied three times a week, with sessions of 60 min. Physical capacities were evaluated with the Senior Fitness Test and HRV was measured with the Polar Team^2^, to obtain parasympathetic indexes SDNN, SD1 and pNN50. The data analysis was performed with the SPSS V.25, with a level of *p* <.05.

**Results**

The comparison between groups showed homogeneity SDNN (EG = 33.52 ± 17.78ms; CG = 33.05 ± 23.22ms) pNN50 (EG = 1.16 ± 2.86%; CG = 1.50 ± 1.68%) SD1 (EG = 10.72 ± 6.34ms; GC = 12.24 ± 5.91ms). In addition, at 6 months significant changes of *p* <.01 were found in SDNN (GE = 45.95 ± 21.67ms; GC = 30.76 ± 15.31ms), pNN50 (GE = 6.57 ± 10.74%; GC = 1.27 ± 2.88%) and *p* <.05 in SD1 (GE = 21.29 ± 18.71ms; GC = 11.93 ± 9.74ms). In EG, there were significant changes (*p* <.01) between baseline measurement and at six months.

**Conclusions**

The multicomponent training program appears to show positive changes in parasympathetic activity in older adults who perform physical activity for a period of six months.

## S13 Mutual support group's management of older people with chronic disease: The importance of fitness levels and active lifestyle promotion

### Edtna Jauregui-Ulloa^1,2^, Julissa Ortiz-Brunel^1^, Esmeralda González-Navarro^2^, Damaris Teresa Tiscareño-Aguilera^1^, Raúl Soria-Rodríguez^1^, Juan R. López y Taylor^1^, Mayra Elizalde-Villarreal^2^, Alberto Ocampo-Chavarria^2^

#### ^1^Centro Universitario de Ciencias de la Salud. Universidad de Guadalajara, Jalisco, México; ^2^Programa de Cardiometabólicas, Medicina Preventiva, Servicios de Salud Jalisco, México

##### **Correspondence:** Edtna Jauregui-Ulloa (edtna.jauregui@hotmail.com)

**Background**

Studies showed that intervention programs that offered strategies at a group level were more likely to improve older people's quality of life and have more beneficial in different dimensions such as social, mental, and physical health compared with one-to-one interventions. The purposes of this study were to evaluate the relationship of healthy-lifestyles behaviors with physical fitness in older people and evaluate the strategies promoting physical fitness.

**Material and Methods**

It was a descriptive mixed design study (qualitative and quantitative approach). Participants aged 60 years and older from different Mutual support groups of the State Health System of Jalisco, México. Rickli-Jone's physical fitness test and interviews with focal groups were applied. Correlation between fitness and lifestyle parameters were defined. Dimensions of reasons for older people to attend the groups were explored.

**Results**

44 female participants of 60 years and older were evaluated. The mean age was 69 years. The most common chronic diseases reported were Hypertension (27.95%), Osteoporosis (37.2%), Osteoarthritis (20.9%) Diabetes Mellitus (9.3%). The profile of muscular strength was between normal ranges and below the normal ranges in flexibility. The strength of patients had a positive correlation with sleeping time (right side p=0.04, left side: p=0.02.) and negative with age (p=<0.06). The Mutual Support Group strategies did not show within the context of a theoretical basis for promoting healthy, active lifestyles. The dose-response of exercise with patients was found to be not well defined. The patient's reasons for attending the groups were centered on personal dimensions (63.63%) and social dimensions (36.3%).

**Conclusions**

Physical activity **s**trategies should be based on patient fitness levels and their chronic disease's clinical state to improve their low physical performance. The Lifestyle approach should include sleeping time, sitting, and walking time to promote healthy fitness.

## S14 Lupus erythematosus and sleep hygiene: A salutogenic perspective

### Jorge Manuel Molina Aguilar (jmolina@uca.edu.sv)

#### Universidad Centroamericana José Simeón Cañas, El Salvador, San Salvador, Centroamérica

**Background**

Research on individuals with lupus erythematosus has a number of limitations: (a) lack of clear theoretical framework of the social mechanisms underlying the development of life skills needed for healthy living, (b) narrow focus centered on biomedical models and health medicalization, and (c) implicit and explicit health hierarchies, especially between social sciences and health sciences. Research on lupus emphasizes the adverse consequences of prescribed medications and treatments. Ambiguous diagnoses and prognoses, and agency capacity of patients and families are emerging subjects in the literature. This research proposes a new theoretical approach to the study of chronic, slow progressing, “stigmatizing” and rare diseases like lupus, based on Aaron Antonovsky’s salutogenic health model (1979, 1987).

**Materials and Methods**

Nine unstructured interviews were conducted using an instrument designed based on the three thematic axis of the Sense of Coherence salutogenic health model. Participants also completed an interview on sleep hygiene. Interviews were conducted online during the COVID-19 quarantine period in El Salvador. Mean age: 53.

**Results** Application of the salutogenic health model resulted in the following contributions: 1) identification of the mechanisms that underlie the daily lifestyle activities of people with lupus (understandability, manageability and significance), 2) recognition that sleep difficulties arise from the interaction between: physical pain, stressors related to health and financial conditions, as well as ability to understand the disease, uncertainty and fear about their social context, 3) alcohol use and self-medication habits as strategies to fall asleep, regardless of prior knowledge of the mental and physical health consequences.

**Conclusion**

Based on this analysis, recommendations are made on how to improve sleep hygiene for individuals with lupus using non-pharmacological methods: a) education as to good sleep hygiene habits, b) monitoring specific cases, c) low impact exercise, d) psychosocial assistance and counseling.

## S15 Quality of life during COVID-19 pandemic in cardiology clinic patients

### Kirby Gutiérrez Arce^1^, Jhonatan Betancourt Peña^2^, Cristian Yovany Rojas Aboyte^1^, Francisco Javier Obeso Sandoval^3^, Maria Cristina Enríquez Reyna^4^

#### ^1^Universidad Autónoma de Occidente, Unidad Regional Guasave, Sinaloa, México; ^2^Escuela Nacional del Deporte, Universidad del Valle, Cali, Colombia; ^3^Instituto Mexicano del Seguro Social, Guasave, Sinaloa, México; ^4^Universidad Autónoma de Nuevo León, Nuevo León, México

##### **Correspondence:** Maria Cristina Enríquez Reyna (maria.enriquezryn@uanl.edu.mx)

**Background**

Pre-existing cardiovascular disease increases vulnerability to serious complications during the COVID-19 pandemic. It is useful to identify the dimensions of quality of life affected in this population to provide preventive care and to support mental health. It was proposed to analyze the quality of life of patients who attend the cardiology consultation.

**Materials and methods**

Descriptive correlational study in cardiology consultation attendees of a private hospital in Sinaloa, Mexico in April 2020. The Spanish version of the WHOQOL BREF questionnaire was applied. This questionnaire distinguishes the dimensions of physical, psychological, social, and environmental health. Analysis with descriptive statistics and Pearson's correlation tests due to the normal distribution of the data (*p* > .05). Reliability and validity were estimated using Cronbach's Alpha and KMO coefficient.

**Results**

Data of 37 participants with an average age of 66 years (*SD* = 14.34; 45.9% women, 54.1% men) are presented. Sixty four percent reported that their quality of life is moderately good. Only 29.7% reported being satisfied with their health. The average raw score of the sample was 76.37 (*SD* = 10.43), when dividing the sample by sex, higher values ​​were found for men than for women (77.69 *vs* 74.83). A positive association was found between age and the perception of environmental health (*r* = .339, *p* <.05). When comparing by sex, this association remained significant only for men (*r* = .548, *p* <.05). The most affected environmental health items refer to purchasing power, possibility of leisure and physical environment (response *range* = 2.03-3.11).

**Conclusions**

In this sample of the cardiology consultation women had lower perceptions of quality of life than men; and age showed an association with the perception of environmental health. The safety of environmental facilities should be increased during the medical care of this type of patients.

## S16 Changes in stereotypes and perceptions towards old age of university students

### Maria Cristina Enriquez Reyna^1^, Lourdes Lizbeth Rocha Aguirre^2^, Nora de la Fuente de la Torre^2^, Perla Lizeth Hernández Cortés^1^

#### ^1^Universidad Autonoma de Nuevo Leon, Nuevo León, Mexico; ^2^Universidad Autonoma de Zacatecas, Zacatecas, Mexico

##### **Correspondence:** Maria Cristina Enriquez Reyna (maria.enriquezryn@uanl.edu.mx)

**Background**

College preparation can be helpful in reducing stereotypes and negative attitudes towards old age. It was proposed to analyze the changes in stereotypes and perceptions towards old age of university students after participating in a curricular class on health care for the older adults.

**Materials and methods**

This cohort design study with two measurements surveyed 69 health sciences students enrolled in an older adult population study class of a public university of Mexico. Stereotypes towards old age were measured with the Kogan's Attitude Towards Old People Scale. The dimensional perception regarding female and male aging was estimated with the Osgood Semantic Differential Scale. Both scales were applied using the validated Spanish version. An online survey was sent in the first and the last day of curricular classes during the first semester of 2020. Differences between cohorts and by type of female or male aging were estimated with the Wilcoxon test.

**Results**

Fifty-five women and 14 men aged 21.67 years (*SD* = 1.33) completed participation in the study. Most of the participants held positive stereotypes towards ageing in the two measurements. Positive differences were detected between first and second cohort stereotypes (*p* < .02). For the second cohort, the perception of male aging changed in the categories of healthy-sickly, fragile-resistant, skillful-clumsy, and integrated-marginalized (*p* < .032). Regarding female aging, differences were observed in the categories of healthy-sickly, skillful-clumsy, integrated-marginalized, and valued-undervalued (*p* < .041).

**Conclusions**

Despite having positive attitudes towards aging, participation in a curricular class on health care for the older adults’ population can lead to changes in stereotypes towards aging. In this sample, the healthy-unhealthy, clever-clumsy, and integrated-marginalized perceptions regarding female and male aging changed after university students participated in a curricular class. These and other aspects should be considered when planning educational programs.

## S17 Symptoms of depression in the older adults during the COVID-19 pandemic. Proposal for physical exercise intervention through Facebook

### Manuel Octavio López-Camacho^1^, Arturo Alonso Cuevas López^1^, Silvia Carolina Medrano Mena^2^, Blanca Rocío Rangel Colmenero^2^, Rosa María Cruz-Castruita^2^

#### ^1^Departamento de Ciencias Sociales y Humanidades, Universidad Autónoma de Occidente, Sinaloa, México; ^2^Facultad de Organización Deportiva, Universidad Autónoma de Nuevo León, Nuevo León, México

##### **Correspondence:** Manuel Octavio López-Camacho (tayo_080386@hotmail.com)

**Background**

Social isolation in the older adults derived from the COVID-19 pandemic is a public health problem because it increases the risk of presenting physical, social and mental health problems such as anxiety and depression. The use of information and communications technology (ICT) can generate social support networks to combat the problems caused by social isolation. The World Health Organization (WHO; 2020) considers that in the face of the pandemic, regular physical activity is necessary to improve mental health. The objective is to evaluate the symptoms of depression in older adults in the state of Sinaloa, Mexico during the social isolation caused by the COVID-19 pandemic and present a proposal for physical exercise intervention through Facebook.

**Materials and methods**

Cross-sectional study, data collected through the Beck Depression Inventory second edition (BDI-II) online, in a preliminary sample of 62 older adults (32 men and 30 women) aged 60 to 83 years, sampling it was for snowball.

**Results**

30% of the older adults showed minimally depressed symptoms, 19% mildly depressed, 26% moderately depressed and 24% severely depressed. In relation to the proposal, a physical exercise intervention is suggested through Facebook, with sessions three times a week of 50 minutes, divided into warm-up, main part and relaxation, focused on resistance, agility, flexibility and balance, the transmission will be live or participants will have access to the videos and will be individually monitored by WhatsApp

**Conclusions**

The high prevalence of older adults with the presence of moderately and severely depressive symptoms shows the need to address this problem, therefore the need arises to create options for non-pharmacological interventions, through the use of ICT to respect the isolation indications Social.

## S18 Physical Activity and Psychosocial Health in Older Adults Confined by COVID-19

### Norma Angélica Borbón-Castro^1^, Andrés Aquilino Castro-Zamora^2^, Rosa María Cruz-Castruita^3^

#### ^1^Educational program degree in sports training, Sonora State University. Hermosillo, Sonora, Mexico; ^2^Educational program degree in human nutrition, Sonora State University. Hermosillo, Sonora, Mexico; ^3^Autonomous University of Nuevo Leon, Faculty of Sports Organization. San Nicolás de los Garza, Nuevo León, México

##### **Correspondence:** Norma Angélica Borbón-Castro (normaa.borbon@ues.mx)

**Background**

The scientific evidence released indicates that COVID-19 develops a more severe clinical picture in the elderly population, particularly in those with pre-existing medical conditions. This is due to as changes in immune response, which exposes the elderly to increased infectivity and a severe form of disease COVID-19. In this context, isolation by prolonged periods of time can cause an adverse response in the welfare of the elderly, for such, the present study aims to analyze the influence of the confinement COVID-19 in the level of physical activity and psychosocial health of older adults.

**Materials and methods**

Systematic review of scientific articles in compliance with the PRISMA statement. A literature search in PubMed, Web of Science, Scielo, CONRICYT, BioMed, ProQuest and Google Scholar databases was performed. Documentary sources were placed on web pages of international health agencies.

**Results**

n = 82 articles included in this systematic review. The social distancing caused by the pandemic has contributed to an increase in sedentary behaviors and has led to changes in lifestyle due to the modification of the type, quantity, intensity and frequency of physical activity, as well as the adoption of eating and irregular sleeping patterns. Easy access to related information to the COVID-19 can generate panic and lack of interaction to share their concerns with family and friends gives them anxiety, boredom and frustration. In addition to these situations are the health measures to face the crisis which have violated the rights of the elderly and affected the little geriatric culture and the trust established between governments, health systems and the elderly, since they exist in the latter uncertainty in decision-making based on age for access to services.

**Conclusions**

The implementation of health promotion programs that include multicomponent physical activity can represent a positive impact on the biopsychosocial health of elderly.

## S19 An overview of a cohort of HIV infected elderly patients in Mexico, the risk factors for frailty syndrome

### Raúl Soria-Rodríguez^1,2^, Ruth Jimenez-Ibarra^1,2^, Javier Méndez-Magaña^1^, Edtna Jauregui-Ulloa^1^, Juan R. López y Taylor^1^, Jaime F. Andrade-Villanueva^1,2^, Luz A. González Hernández^1,2^

#### ^1^Universidad de Guadalajara, Centro Universitario de Ciencias de la Salud; Guadalajara Jalisco, México; ^2^Unidad de VIH, Hospital Civil Fray Antonio Alcalde, Guadalajara Jalisco, México

##### **Correspondence:** Raúl Soria-Rodríguez (raul.soria@academicos.udg.mx)

**Background**

Life expectancy on HIV-infected subjects has significantly improved since the introduction of antiretroviral therapy; thus, the HIV-infected population is becoming older. Some known risk factors for frailty include chronic kidney disease, cerebrovascular disease, cardiovascular disease, smoking, and high and low BMI, while increased exercise has a protective association with frailty. Studies suggest that the prevalence of frailty is higher in HIV older people compared to the general population. Nevertheless, information regarding the elderly-infected population is still limited.

**Materials and methods**

Data were retrospectively obtained from an electronic database at the HIV-unit in Hospital-Civil de Guadalajara. All patients ≥50 years being actively followed-up at the site by February 2019 were included. Comparisons between male and female groups were performed with T-student and X^2^ tests. This study aimed to describe some risk factors for frailty in a cohort of patients ≥50 years. The significance level was established at ≤5%.

**Results**

A total of 475 patients ≥50 years being actively followed-up were detected. This is 17% of the total population in this database. The average age was 57.4 years old, and it ranged from 50– 90 years. Most patients were male (82%). BMI (≥25) was higher in women than in men (p=<0.019). On the other hand, the smoking index was higher in men than in women (p=<0.001). Concerning comorbidities, there was a higher prevalence of arterial hypertension (p=<0.014) and depression (p=<0.001) in women compared with men, with a significantly higher VACS index.

**Conclusions**

The higher BMI and the association of the VACS index to markers of chronic inflammation and hypercoagulability may pose special risks for cardiovascular diseases and frailty in men and women, and men's tobacco use is an increased risk yet. Unfortunately, there is no physical activity (PA) program for these patients; that is why we couldn't evaluate PA or sedentarism. In the future, the purpose is to include the implementation of early interventions like PA programs to decrease and modify the frailty risk factors.

## S20 Is it endomorphy determination equally effective in men and women?

### Fernando Alacid^1,4^, Oriol Abellán-Aynés^2,4^, Luis Manuel Martínez-Aranda^3,4^, Daniel López-Plaza^2,4^

#### ^1^Department of Education. Health Research Centre, University of Almería, Spain; ^2^Sport Medicine Chair, Catholic University of San Antonio, Murcia, Spain^; 3^Faculty of Sport, Neuroscience of Human Movement Research Group (Neuromove), Catholic University of San Antonio, Murcia, Spain; ^4^Ibero-American Network of Researchers in Applied Anthropometry

##### **Correspondence:** Fernando Alacid (falacid@ual.es)

**Background**

The Heath-Carter somatotype method has been widely used to identify the morphological profile in different sports disciplines. Sex differences in somatotypes appeared to be stronger for endomorphy, with larger values in women. This is related with higher values of total fat mass and skinfolds in women. Marini et al, showed that calf, triceps and midthigh skinfolds were greater in females compared to males in the 100.0%, 98.2% and 90.5% of the studied cases, respectively. Moreover, some studies have highlighted the importance of the lower limb skinfolds in the estimation of body adiposity. However, only three upper body skinfolds are used to calculate endomorphy (triceps, subscapular and supraspinal). Thus, endomorphy might be undervalued in women. The aim of this study was to compare the sex differences in endomorphy related with total adiposity estimated by skinfolds.

**Materials and methods**

Fifty young kayakers (25 women: 15.5±0.5 years-old, body mass: 59.9±6.9 kg, stature: 165.2±6.5 cm; 25 men: 15.6±0.6 years-old, body mass: 69.1±10.0 kg, stature: 174.6±5.4 cm) participated in the study. They were assessed with a battery of 32 anthropometric dimensions. Endomorphy was calculated using the Heath-Carter equations^1^. Total adiposity was determined from sums of six and eight skinfolds and body fat percentage^6,7^. Pearson’s correlation coefficient (*r*) was used to determine the interrelationships between endomorphy and the rest of variables.

**Results**

Endomorphy was significantly associated with fat mass equations and sums of skinfolds (*p*≤0.05). The *r* values ranged from 0.603 to 0.971 and were always higher in men than in women (∑6 skinfolds: 0.957-0.909; ∑8 skinfolds: 0.971-0.790; %Fat-Mass-Slaughter^6^: 0.851-0.603; %Fat-Mass-Kerr^7^: 0.906-0.774).

**Conclusions**

The difference of body fat distribution in men and women and the limited number of upper body skinfolds used in the calculation of endomorphy might indicate a lower relationship with total adiposity and a probable underestimation of this variable in

## S21 Relationship between physical activity habits and maximum oxygen volume performance in adult women

### Salvador Baena-Morales^1^, Francisco Tomás González-Fernández ^2^, Daniel Ramos-Pérez^3^, Honorato Morente-Oria^4^

#### ^1^Department of general and specific didactic, Faculty of Education, University of Alicante, Alicante, Spain; ^2^Department of Physical Activity and Sports Sciences, Pontifical University of Comillas, CESAG, Mallorca, Spain; ^3^University of Malaga, Malaga, Spain; ^4^Didactic of Languages, Arts and Sport’s Department, University of Málaga, Málaga, Spain

##### **Correspondence:** Salvador Baena-Morales (salvador.baena@ua.es)

**Background**

Traditionally women have been less active and physically active than men. Although the recent literature continues to indicate this gender gap, the difference between the sexes when practicing sport seems to be decreasing. The reasons for these differences are multifactorial, with a number of intrinsic and extrinsic motivations indicated as requiring further research. Self-perception in physical performance has been documented as one of the reasons that condition women to perform exercise and physical activity. Therefore, the aim of this research was to identify and relate the self-perception and motivations of healthy adult women to performance in a VO2max test.

**Materials and methods**

A total of 31 women students of the University of Granada (Spain) (years=21.12 ± 2.01), (BMI= 21.85 ± 2.38) completed the research. The study was divided into two main tests. On the one hand, the measurement of a sub-maximal incremental stress test following established ASCM indications (maximum power=90.15 ± 21.34 watios). Heart rate and ventilatory parameters were measured during the stress test using a metabolic measurement system and a gas analyzer (UVA=20.56 ± 3.61 ml_/_kg_/_min). At the end of the test, the study sample completed the self-completed Healthy Lifestyle Questionnaire and the International Short Form Physical Activity Questionnaire (IPAQ-SF) and the Self Report of Reasons for Physical Activity (AMPEF).

**Results**

The descriptive statistics of the AMPEF questionnaire were correlated with VO2Max. A positive relationship was detected between VO2Max and the body image (PIC) (r=0.001)

**Conclusions**

The results obtained help to confirm the importance of a positive self-perception in women regarding their weight and body image for a better physical performance. Although the data found cannot be generalized, they establish a potential relationship between better physical performance and self-perception in the best adults.

## S22 HIIT does not produce chronic stress on the Autonomous Nervous System in sedentary adults

### Isaac García-Flores, Jorge A. Aburto-Corona, Melinna Ortiz-Ortiz, Luis Mario Gómez-Miranda

#### Sports Faculty, Autonomous University of Baja California, Baja California, México

##### **Correspondence:** Luis Mario Gómez-Miranda (lgomez8@uabc.edu.mx)

**Background**

HIIT is a widely used method today. In the scientific literature, cardiovascular improvements are reported from their first sessions in different populations, however, the chronic stress that could cause has not been reported, therefore, **t**he objective was to determine whether high intensity interval training (HIIT) presents stress or fatigue values on the Autonomous Nervous System (ANS) through Heart Rate Variability (HRV)​​, among apparently healthy sedentary adults.

**Materials and methods**

Twenty sedentary adults (age=33.3±5.2 years) participated in a treadmill HIIT program for five weeks (three sessions per week). Maximum aerobic velocity (MAV) was estimated by the 30-15 IFT field test. Resting HRV was measured using a heart rate monitor (Polar® RS800CX). HRV was monitored through the RMSSD variable prior to HIIT training in sessions 1, 4, 8, 12 and 16. The HIIT program load was measured by VAM, starting with a three-minute warm-up (40% VAM), five one-minute intervals (80% VAM) with active one-minute rest between intervals (50% VAM) and, finally, five minutes of recovery (40% VAM).

**Results**

No significant difference was found in the simple effect of measurements for RMSSD (p = 0.14; η2 = 0.08).

**Conclusions**

The application of 16 HIIT sessions with 48-hour breaks between them does not present alterations on the ANS measured through HRV with the RMSSD variable, therefore, this type of training does not cause stress or overload in sedentary population.

## S23 Sustained attention after 40 minutes of physical exercise at 75% of ventilatory threshold on university students

### Francisco Tomás González-Fernández^1^ and Luis Manuel Martínez-Aranda^2^

#### ^1^Red Iberoamericana de Investigación en Antropometría Aplicada, Department of Physical Activity and Sport Sciences, CESAG, Comillas Pontifical University, Mallorca, Spain; ^2^Red Iberoamericana de Investigación en Antropometría Aplicada, Faculty of Sport, Neuroscience of Human Movement Research Group (Neuromove), Catholic University of San Antonio, Murcia, Spain

##### **Correspondence:** Francisco Tomás González-Fernández (francis.gonzalez.fernandez@gmail.com)

**Background**

Sustained attention is the capacity to focus on an activity during a long period of time to respond appropriately to relevant stimuli. In fact, this capacity is moderated by exercise and duration and, therefore, performing exercise before many day life activities may improve the sustained attention in order to keep performance at its best.

**Materials and methods**

A group of 36 healthy students participated in the present study. First, their Ventilatory Threshold (VT) was obtained using a submaximal incremental effort test. Second, psychomotor vigilance task (PVT) was performed as a reliable instrument to measure sustained attention, for 10’ in two sessions: 1) after of 40’ at 75% of Ventilatory Threshold, and 2) after of 40’ sitting reading magazines.

**Results**

In order to investigate participants’ Reaction Times (RT), a repeated measures analysis of variance (ANOVA) using mean RT data including the factors of Session (after of 40’ of exercise, after of 40’ sitting) and Time on task (10’) revealed that participants were significantly faster after 40’ at 75% of VAT than after 40’ sitting (control session). In addition, this RT improvement was found for the total duration of the task.

**Conclusions**

In the present research, we investigated how the efficiency of sustained attention can be affected positively after 40’ of moderate aerobic exercise. In this regard, the literature suggests that exercising at moderate intensity produced different physiological changes that have been usually linked to the activation of participants. Therefore, our results demonstrated that sustained attention performance improves after moderate exercises and extends the use of exercise to study the improvement of attention among different groups of workers or students.

## S24 Effect of intermittent load exercise on physiological stress and myogenic leucocytosis in healthy warehouse workers

### Luis Manuel Martínez-Aranda^1^, Francisco Jesús Llorente-Cantarero^2,3,4^, Francisco Tomás González-Fernández^5^

#### ^1^Ibero-American Research Network in Applied Anthropometry, Faculty of Sport, Neuroscience of Human Movement Research Group (Neuromove), Catholic University of San Antonio, Murcia, Spain; ^2^Department of Specific Didactics. Faculty of Education Sciences. University of Cordoba, Cordoba, Spain; ^3^Institute of Biomedical Research (IMIBIC), Cordoba, Spain; ^4^Biomedical Research Centre Network &quot;Physiopathology of Obesity and Nutrition” (CIBEROBN). Madrid, Spain; ^5^Ibero-American Research Network in Applied Anthropometry, Department of Physical Activity and Sport Sciences, CESAG, Comillas Pontifical University, Mallorca, Spain

##### **Correspondence:** Luis Manuel Martínez-Aranda (lmmartinez2@ucam.edu)

**Background**

Vigorous physical activity while carrying variable loads can be found in numerous jobs in our society, without any consideration of the possible repercussions it may have on workers’ health. A specific example of this is the work carried out by warehouse workers. It has been observed that vigorous exercise alters both, the amount and the functional capabilities of many immune cell types, causing alterations in local and systemic levels of several molecular mediators of the immune system. The level of leucocytosis appears to be related to several variables, including the degree of stress experienced by the individual. The objective of this study was to determine the physiological effects of the physical stress generated after an intermittent load exercise, simulating an approximate working day.

**Material and Methods**

The sample consisted on 26 healthy subjects, randomly distributed into two groups: a control group (n = 13; 25.38 ± 3.09 years old) and the experimental group (n = 13; 25.61 ± 7.04 years old). Anthropometric and hemodynamic values, as well as a complete biochemistry were measured before and after the intermittent load exercise. During the exercise, the number of loads, the carried weight and the duration of the loads were measured for each subject.

**Results**

There were differences before and after exercise, revealing higher values of leukocytes and neutrophils (P <0.001) and lower lymphocyte (P <0.001) and eosinophils (P = 0.002) post-exercise compared to pre-exercise while monocytes and basophils remained unchanged. Furthermore, there was a prominent increase in post-exercise creatine kinase values (P = 0.003).

**Conclusion**

Our results suggest the involvement of a myogenic leucocytosis process in its second stage, characterized by an increased number of leukocytes in parallel with processes of lymphopenia, eosinopenia and neutrocytosis, as well as an increase in creatine kinase values.

## S25 Biological maturity and predicted adult height in young basketball players based on their playing positions

### Daniel López-Plaza^1,4^, Luis Manuel Martínez-Aranda^3^, Oriol Abellán-Aynés^1,4^, Fernando Alacid^2,4^

#### ^1^Ibero-American Network of Researchers in Applied Anthropometry, Sport Medicine Chair, Catholic University of San Antonio, Murcia, Spain; ^2^Ibero-American Network of Researchers in Applied Anthropometry, Department of Education. Health Research Centre, University of Almería, Spain; ^3^Faculty of Sport, Neuroscience of Human Movement Research Group (Neuromove), Catholic University of San Antonio, Murcia, Spain

##### **Correspondence:** Daniel López-Plaza (dlplaza@ucam.edu)

**Background**

In competitive sports, there are certain physical and anthropometric attributes that play a key role in the consecution of optimal performance. Depending on the characteristics of the sport, discipline or playing position, these attributes are different. In addition, their development during childhood and adolescence is strongly influenced by biological maturity. Traditionally, height and arm spam have been two determinants for talent identification purposes and for playing position designation in basketball. The aim of this study was to compare the percentage of predicted adult height as a measure of maturity in young basketball players based on their playing position.

**Materials and methods**

Twenty nine young basketball players (14.5±0.61 years-old, body mass: 71.45±10.86 kg, height: 183.88.2±8.73 cm; arm span: 188.39±11.43 cm) that trained on regular basis (>3 sessions/week) volunteered in the study. Participants were allocated in two groups depending on their usual playing position (Guards and Forwards-Centers). The prediction of adult height was investigated using 2 equations: Tanner-Whitehouse III^5^ (TW3) and Sherar^6^. For the determination of bone age, an ultrasonography device BAUSTM was used (Sonicbone Medical Ltd, Rishon Lezionel, Israel) while the peak at height velocity (PHV) was determined by the procedures described by Mirwald^2^ using basic anthropometric measurements. The differences between groups (mean) were analysed using t-test for independent variables.

**Results**

Guards revealed significantly lower percentages of predicted adult height than the Forwards-Centers (*p*≤0.05) in both, TW3 method (97.52±3.06 and 101.90±2.49, respectively) and Sherar method (94.84±3.29 and 97.57±1.36, respectively). Moreover, the peak at height velocity (PHV) was significantly higher (*p*≤0.01) in Guards (13.24±0.70) compared to Forwards-Centers (12.59±0.59).

**Conclusions**

Young basketball forwards and centers were characterised by greater maturity status and a lower rate of height development than guards during adolescence. Therefore, biological maturity might be useful not only for the identification of future physical and morphological development levels such as in height but also for improving playing position designation.

## S26 Profiling physical fitness of undergraduate physical culture, sports and recreation students in Colombia using machine learning

### Isabel Sánchez-Rojas^1^, Darío Mendoza-Romero^1^, Jorge L. Petro^2,3^, Yurany Moreno^4^, Diego A. Bonilla^2,3,4,5^

#### ^1^Grupo de Investigación Ciencias Aplicadas al Ejercicio, Deporte y Salud - GICAEDS, Universidad Santo Tomás, 205070 Bogotá, Colombia; ^2^Research Division, DBSS International SAS, 110861 Bogotá, Colombia; ^3^Research Group in Physical Activity, Sports and Health Sciences (GICAFS), Universidad de Córdoba, 230002 Montería, Colombia; ^4^Research Group in Biochemistry and Molecular Biology, Universidad Distrital Francisco José de Caldas, 110311 Bogotá, Colombia; ^5^kDNA Genomics®, University of the Basque Country UPV/EHU, Joxe Mari Korta Research Center, 20018 Donostia-San Sebastián, Spain

##### **Correspondence:** Isabel Sánchez-Rojas (isabel.sanchez@usantotomas.edu.co)

**Background**

The academic curriculum has shown to promote sedentary behavior in university students. The study aimed (first) to characterize the physical fitness of undergraduate students enrolled in a physical activity-related program by the generation of profiles using unsupervised machine learning and (second) to identify differences between sexes, academic years, socioeconomic strata and the generated profiles.

**Materials and methods**

Healthy and physically active undergraduate students (n=542; 19.8±2.2 years; 66.0±10.3 kg; 169.5±7.8 cm) residing in Bogotá participated in this STROBE-based study. To assess physical fitness, we measured indirect VO_2max_ (Cooper and Léger tests), lower-limb power (horizontal jump), sprint (30 m), agility (shuttle run) and flexibility (sit-and-reach). Participants were profiled using k-Medoids (PAM) and hierarchical clustering (HC) after identification of the optimal number of clusters and internal validations with the R packages ‘*NbClust*’ and ‘*Purr*’+‘*clValid*’, respectively. Non-parametric tests were used to identify differences (U-Mann-Whitney and Kruskal-Wallis). Statistical analysis were performed within R v4.0.2 and SPSS v26.

**Results**

Participants had different distribution according to i) sex (82.1% men, 17.8% women); ii) freshman (51.4%), sophomore (24.3%), junior (10.8%) and senior (13.2%) years; and iii) SS 1 (0.3%), 2 (16.4%), 3 (64.0%), 4 (16.6%), 5 (1.8%) and 6 (0.7%). Two profiles (69.9% cluster 1, 30.0% cluster 2) were generated using HC, which showed better performance in comparison to PAM. Matching analysis revealed that sex might partially explain the variation in the data (women 28.8% [cluster 1] and 71.1% [cluster 2]; men 78.8% [cluster 1] and 21.1% [cluster 2]). All variables were significantly different between sexes (excepting BMI) and profiles (excepting body mass and sprint). Seniors and juniors showed better physical fitness than first-years students. No significant differences were found between socioeconomic strata.

**Conclusions**

Applying machine learning to characterize student populations contribute to the identification of patterns besides providing a reproducible and a fast tool to analyze data.

## S27 Clustering-based characterization of body composition and morphology in Colombian nutrition and dietetics undergraduate students (CES University, 2016-2020)

### Diego A. Bonilla^1,2,3,4^, Katherine Franco-Hoyos^1^, Alejandra Agudelo-Martínez^1^, Maximiliano Kammerer-López^1^, Luis Felipe Bedoya-Bedoya^1^, Yurany Moreno^4^, Jorge L. Petro^2,3^

#### ^1^Grupo de investigación Nutral, Facultad Ciencias de la Nutrición y los Alimentos, Universidad CES, 050021 Medellín, Colombia; ^2^ Research Division, DBSS International SAS, 110861 Bogotá, Colombia; ^3^Research Group in Physical Activity, Sports and Health Sciences (GICAFS), Universidad de Córdoba, 230002 Montería, Colombia; ^4^Research Group in Biochemistry and Molecular Biology, Universidad Distrital Francisco José de Caldas, 110311 Bogotá, Colombia

##### **Correspondence:** Diego A. Bonilla (dabonilla@dbss.pro)

**Background**

Unhealthy dietary habits are frequent in college students, which can affect health and academic performance. The aim of this study was twofold: i) to characterize the body composition (BC) and morphological features (MF) of freshman students based on unsupervised machine learning using anthropometric (raw and derived) variables, and ii) to identify the factors influencing differences.

**Materials and methods**

A total of 320 students (278F; 42M; 19.9±3.1 years; 58.8±12.2 kg; 162.0±7.9 cm) enrolled in the nutrition and dietetics program at Universidad CES (2016-2020 cohorts) participated in this cross-sectional study. BC was evaluated using the 5-compartmental model of body fractionation, equations to estimate fat mass and sum of skinfolds, while MF was assessed with raw variables, indices (e.g., cormic, muscle-to-bone, locomotive) and somatotype. The R packages *‘NbClust’* and *‘clValid’* were used to select the best number of clusters and clustering method. The data were analyzed with the Mann-Whitney U and Kruskal-Wallis tests in SPSS.

**Results**

Hierarchical clustering with bottom-up approach (Ward’s method) showed to be the most suitable to analyze our data. Two statistically significant clusters, for almost all variables, were identified (cluster 1 = 227, cluster 2 = 93). These clusters represented students that were: robust, with higher adiposity, endo-mesomorph and less physically active (cluster 1); and with lower adiposity, endo-ectomorph and more physically active (cluster 2). Interestingly, sex explained a high percentage of data variation (224 women in cluster 1). In fact, between-sex differences were obtained in all variables except for age, femur length and axilar skinfold. As expected, physical activity levels showed significant differences in BC and MF. The cohort year did not result in relevant differences in BC or MF.

**Conclusions**

The two phenotypes obtained from this clustering study provides relevant information about BC and MF in first-year students and might help to the CES University interventions.

## S28 Effects of the use of Jatobá (*Hymenaea courbaril*) juice on physical performance related to exercises that require muscle power

### Joy Braga Cavalcante^1^, Rafaella Cristiny da Silva Cavalcante^1^, João Rafael Valentim Silva^1^, Marcel Bahia Lanza,^2^ Carolina Freitas Silva^1^, Romeu Paulo Martins Silva^1,3^

#### ^1^Department of Health and Sport Sciences, Federal University of Acre, Acre, Brazil; ^2^School of Sport, Exercise, and Health Sciences, Loughborough University, Leicestershire, England; ^3^Department of Biotechnology, Federal University of Catalão, Goiás, Brazil

##### **Correspondence:** Romeu Paulo Martins Silva (romeupms@gmail.com)

**Background**

Physical exercise is a muscular activity that generates force and interrupts homeostasis, resulting in metabolic disorders, in which recovery depends on the restoration of muscular glycogen stores, which can be potentiated with the use of ergogenic resources. The aim of the study is determine the effect on the physical performance of young adults related to muscle power exercises after taking the Jatobá **(*Hymenaea courbaril*)** juice.

**Materials and methods**

The sample consisted initially of 60 men finishing with 18 healthy and active men. The participants performed three sessions of physical tests, with an interval of one week each, taking Jatobá juice (GT) and then starch (Placebo Group), fixed bar flexion, soil and abdominal flexion, all of them oriented to maximize of repetitions in 15 seconds. After the first session the participants were instructed to take 50 ml of jatobá wine twice daily GT) for five days after seven days performed the same procedures eating only starch where participants did not know what was being ingested (Placebo Group). Blood samples were collected before and immediately after the physical test sessions for the biochemical determinations.

**Results**

There was a change in the mean number of repetitions in all the exercises proposed for a p <0.05 when compared to the placebo group and the one that used the juice. When comparing the before and after enzyme concentrations in the different tests, plasma creatine kinase (CK-NAC) values ​​were higher in post-workout at p <0.05 in the Placebo Group. Lactate dehydrogenase (LDH), aspartate aminotransferase and (AST) alanine aminotransferase (ALT) showed no difference at p <0.05.

**Conclusion**

Juice of Jatobá in the dosage used in this research proved to be effective in improving physical performance in the proposed exercises, with variation in the enzyme that causes greater muscle damage.

